# Low levels of very-long-chain *n*-3 PUFA in Atlantic salmon (*Salmo salar*) diet reduce fish robustness under challenging conditions in sea cages

**DOI:** 10.1017/jns.2017.28

**Published:** 2017-06-28

**Authors:** Marta Bou, Gerd M. Berge, Grete Baeverfjord, Trygve Sigholt, Tone-Kari Østbye, Bente Ruyter

**Affiliations:** 1 Nofima (Norwegian Institute of Food, Fisheries, and Aquaculture Research), PO Box 210, N-1432 Ås, Norway; 2 Department of Animal and Aquacultural Sciences, Norwegian University of Life Sciences, N-1430 Ås, Norway; 3 Nofima, N-6600 Sunndalsøra, Norway; 4 BioMar AS, N-7484 Trondheim, Norway

**Keywords:** Aquafeed, DHA, EPA, Essential fatty acids, Fish nutritional requirements, ARA, arachidonic acid, EFA, essential fatty acid, NL, neutral lipid, PC, phosphatidylcholine, PE, phosphatidylethanolamine, PI, phosphatidylinositol, PL, phospholipid, PS, phosphatidylserine, VLC, very-long-chain, VO, vegetable oil

## Abstract

The present study aimed to determine the minimum requirements of the essential *n*-3 fatty acids EPA and DHA in Atlantic salmon (*Salmo salar*) that can secure their health under challenging conditions in sea cages. Individually tagged Atlantic salmon were fed 2, 10 and 17 g/kg of EPA + DHA from 400 g until slaughter size (about 3·5 kg). The experimental fish reared in sea cages were subjected to the challenging conditions typically experienced under commercial production. Salmon receiving the lowest EPA + DHA levels showed lower growth rates in the earlier life stages, but no significant difference in final weights at slaughter. The fatty acid composition of various tissues and organs had remarkably changed. The decreased EPA + DHA in the different tissue membrane phospholipids were typically replaced by pro-inflammatory *n*-6 fatty acids, most markedly in the skin. The EPA + DHA levels were maintained at a higher level in the liver and erythrocytes than in the muscle, intestine and skin. After delousing at high water temperatures, the mortality rates were 63, 52 and 16 % in the salmon fed 2, 10 and 17 g/kg EPA + DHA. Low EPA + DHA levels also increased the liver, intestinal and visceral fat amount, reduced intervertebral space and caused mid-intestinal hyper-vacuolisation. Thus, 10 g/kg EPA + DHA in the Atlantic salmon diet, a level previously regarded as sufficient, was found to be too low to maintain fish health under demanding environmental conditions in sea cages.

During the last decades, marine raw ingredient scarcity and sustainability concerns have caused major compositional changes in the commercial salmon feeds in Norway, from essentially a marine-based diet in the early 1990s to a diet with 70 % plant ingredients at present^(^^1^^)^. The dietary fatty acid profile is one of the main factors determining the fatty acid composition of farmed fish. Therefore, a dietary increase in typical plant fatty acids and a concomitant reduction in EPA (20 : 5*n*-3) and DHA (22 : 6*n*-3) are reflected remarkably in the body lipid composition of fish fed these diets. The dietary requirement of *n*-3 fatty acids α-linolenic acid (18 : 3*n*-3), EPA and DHA of salmonids has been reported to range from 5 to 10 g/kg feed depending on experimental conditions (for a review, see Glencross^(^^2^^)^). In most of these studies, the requirement was set for relatively small fish in short-term trials on land tanks, and the requirements were primarily decided based on fish growth and survival. In a recent trial from our group, the requirement of essential fatty acids (EFA) was reassessed in salmon (from 40 to 400 g) fed a fish meal and fish oil-free diet supplemented with 0, 5, 10, 15 or 20 g/kg feed of EPA, DHA or the combination of these fatty acids in a 1:1 ratio in sea water^(^^3^^)^. Although mortalities were not noted, fish fed 0 g/kg diet showed a moderate decrease in growth compared with those fed the EPA and DHA-supplemented diets. In addition, alterations in the fatty acid composition of organs and tissues, particularly in the phospholipid (PL) fraction, in response to the lack of *n*-3 very-long-chain (VLC)-PUFA were detected, with a major increase in *n*-6 fatty acids and presence of hyper-vacuolisation and swollen enterocytes in the midgut, indicating that the changes in membrane PL might influence tissue integrity and function.

Membrane PL are the source of VLC-PUFA substrates required for the synthesis of eicosanoids, particularly arachidonic acid (ARA; 20 : 4*n*-6). The *n*-6 fatty acids are known to promote the formation of pro-inflammatory and pro-aggregatory eicosanoids, whereas *n*-3 fatty acids have the opposite effects. Variable results concerning the health impact of increased levels of *n*-6 fatty acids and reduced *n*-3 fatty acids in salmonids have been reported, ranging from no apparent adverse effects^(^^4^^,^^5^^)^ to impaired macrophage function^(^^6^^)^, lower leucocyte production^(^^7^^)^, or decreased fish resistance to infection^(^^8^^)^. This indicates that understanding how low dietary EPA and DHA levels influence the composition, function and health of specific tissues is essential.

Lipids and fatty acids have important biological functions in fish. In particular, tissue *n*-3 EPA and DHA and *n*-6 ARA levels play a key role in maintaining fish health by participating in different processes such as cell synthesis, ontogenesis, endocrine and immune system function and control, pigmentation, and neural tissue function and development^(^^2^^,^^9^^)^. Similar to salmon, humans have also undergone major nutritional changes over the last few decades; at present, in the Western diet, the *n*-6:*n*-3 ratio is increased and ranges from 10:1 to 20:1^(^^10^^)^. This is paralleled by an increased prevalence of diseases involving inflammatory processes, such as CVD, obesity, inflammatory bowel disease, rheumatoid arthritis and cancer^(^^11^^)^. Ongoing changes in lifestyle and diet are known to be related to epidemic chronic degenerative diseases in Western countries^(^^12^^)^. Whether Atlantic salmon (*Salmo salar*) can develop lifestyle-related diseases if they are fed very low EFA levels during their life cycle is not yet known.

Few studies have assessed the long-term effects of feeding Atlantic salmon low EPA and DHA levels during their production cycle. Recently, the dietary EPA and DHA requirement of Atlantic salmon was found to be above 2·7 % of fatty acids (10 g/kg feed) for optimal long-term growth in sea water^(^^13^^)^. However, they found no effect on mortality when the dietary EPA and DHA level was reduced to 1·4 % of fatty acids (4 g/kg feed). Nonetheless, this trial was conducted under controlled experimental conditions, which do not reflect the fluctuating environmental conditions that salmon experience in commercial aquaculture and where an accumulated loss through the entire production cycle generally ranges from 15 to 20 %^(^^14^^)^. Another experiment performed under commercial production conditions showed that reducing dietary EPA and DHA from 8 to 5 % of fatty acids (from 26 to 16 g/kg feed) during the production cycle in sea water did not affect Atlantic salmon growth, health or product quality^(^^15^^)^. Nevertheless, a dietary content of 16 g/kg feed of EPA and DHA is close to the dietary inclusion levels in commercial salmon diets used at present. Future growth in aquaculture production might require further dietary reduction from the approximately 17 g/kg EPA + DHA levels in the present commercial feeds for salmon. However, a decrease below the tolerable fish limits in these EFA might have profound effects on fish health and thus might cause important economic losses for the farming industry. Therefore, determining the lower dietary requirement of Atlantic salmon for EPA and DHA, and whether environmental conditions and stress influence this requirement, is necessary.

The present study aimed to determine the dietary requirement of EPA and DHA in Atlantic salmon diets in order to prevent nutritional pathologies and secure fish growth and robustness throughout the production cycle under fluctuating environmental conditions similar to those experienced in commercial production.

## Materials and methods

### Rearing facilities and experimental design

The feeding trial was performed using 450 individually tagged (passive integrated transponder tags; Biosonic) Atlantic salmon from fourteen pre-dietary groups that had been fed different dietary levels of EPA and/or DHA in early life-stages. Fish (approximately 1·2 kg) were evenly distributed into three sea cages of 25 m^2^ area at the LetSea Aquaculture Research Station in Dønna, Norway (February 2014). Fish in each cage were provided one of the three experimental diets with low (2 g/kg feed), intermediate (10 g/kg feed), and formulation resembling a commercial diet (17 g/kg) of EPA and DHA. Feeds were produced as 4, 5 and 7 mm pellets according to fish size. The fish were reared under standard farming conditions. Temperature, salinity and oxygen were recorded every 15 min. From February to April, the water temperature was maintained around 5°C. It was then gradually increased until the end of July, when the highest water temperature was recorded (17·5°C); thereafter, it was gradually decreased to 9°C by mid-November, when the experiment ended. Mortalities were recorded throughout the experiment. The fish were treated for sea lice four times by providing chemical bath treatment with azamethiphos (0·2 ml/m^3^ for 35 min; Trident Vet, Neptune Pharma) and deltamethrin (0·3 ml/m^3^ for 30 min; ALPHA MAX, PHARMAQ) during the feeding trial (25 April, 10 July, 20 August and 6 November 2014) following the standard procedures of LetSea Research Station.

#### Pre-feeding history of Atlantic salmon before trial in sea cages

From 40 to 400 g fish were fed one of the fourteen diets: thirteen experimental diets formulated to test five dietary levels of EPA, DHA, or a 1:1 mixture of EPA and DHA (0, 5, 10, 15 and 20 g/kg feed in each dietary group) and a control diet with a formulation resembling a commercial diet. A detailed description of the experimental conditions and dietary composition is provided by Bou *et al.*^(^^3^^)^. Thereafter, the individually tagged fish from all pre-dietary groups were evenly distributed into nine tanks on land and fed the three experimental diets containing different levels of EPA and DHA (2, 10 and 17 g/kg feed) until they reached 1·2 kg body weight and were transported to the sea cages.

### Feeds and feed production

The experimental diets were produced by BioMar AS. Diets were distributed in excess (approximately 10 %) of the expected feed intake to ensure feeding to satiation by using automatic feeders. The fish were fed two meals per d when the water temperature was above 8·0°C, and one meal per d at water temperatures below 8·0°C. Feed intake per cage was recorded weekly based on the daily feed ration and corrected by waste feed pellets collected using a LiftUp system. The formulation and chemical composition of the experimental diets are shown in Table 1. A small percentage of krill meal was added to all diets as an appetite enhancer. The chemical composition of the diets was determined by proximate composition analysis according to previously described standard methods^(^^16^^)^. The fatty acid compositions of the diets, which were determined using the method described below, are provided in Table 2. The experimental diets contained about 32·8 % fat and 36·5 % protein. The 2 g/kg EPA + DHA diet was fishmeal- and fish oil-free, and the main oil source was a mixture of poultry, rapeseed and linseed oil (50:30:20; by vol.), and the main protein sources were poultry meal and soya protein concentrate. The protein sources of the 10 g/kg EPA + DHA diet were the same as those from the 2 g/kg diet, but fish oil was added at the expense of poultry oil and rapeseed oil to provide the dietary levels of EPA and DHA. The composition of the 17 g/kg EPA + DHA diet resembled a commercial one where the main protein sources are fish meal and soya protein concentrate and fish oil and rapeseed oil as the main lipid sources. Despite the different protein sources used in the experimental diets, the amino acids were carefully balanced to avoid variations (Supplementary Table S1). The Atlantic salmon were fed the three experimental diets from 26 weeks after smolt transfer (approximately 400 g; October 2013) until harvest (approximately 3·5 kg; November 2014), although in this article only the results for the period in sea cages are reported. The dietary groups are named according to their EPA and DHA contents in the feed as 2, 10 and 17 g/kg feed, corresponding to EPA and DHA dietary levels of 0·7, 3·5 and 5·7 % of total fatty acids, respectively.

**Table 1.** Ingredients and proximate composition of the experimental diets (7 mm)

	2 g/kg EPA + DHA	10 g/kg EPA + DHA	17 g/kg EPA + DHA
Formulation (%)			
Poultry meal*	18·00	18·00	–
Fish meal†	–	–	15·00
Krill meal‡	3·00	3·00	3·00
Poultry oil*	15·00	6·51	–
Rapeseed oil§	7·50	5·00	9·83
Linseed oil‖	5·00	5·00	–
Fish oil¶	–	11·08	17·19
Soya protein concentrate	24·97	25·00	25·00
Wheat	11·20	10·81	12·00
Wheat gluten	–	–	1·00
Maize gluten	5·00	5·00	7·00
Pea protein	5·14	6·15	–
Horse beans dehulled	–	–	8·05
Monocalcium phosphate	1·24	1·21	1·90
Amino acids	1·09	0·44	0·83
Vitamin C, 35 %	–	–	0·06
Vitamin E, 50 %	–	–	0·03
Process additives	0·33	0·33	0·28
Ethoxyquin 66·6 %, dry	0·02	0·02	–
Lucatin pink	0·04	0·04	0·04
Vitamin and minerals	–	–	0·40
Vitamin NOFIMA	2·00	2·00	–
Mineral NOFIMA	0·52	0·52	–
Yttrium	0·05	0·05	0·05
Chemical composition			
DM (%)	95·2	96·3	95·3
Fat (%)	32·4	34·5	31·6
Proteins (%)	37·3	36·1	36·2
Ash (%)	6·0	6·1	6·4
Gross energy (MJ/kg)	24·3	24·4	23·8

* GePro.

† Mix of North Atlantic and South American fish meal.

‡ Aker BioMarine.

§ Rapeseed oil, crude.

‖ Linseed oil, crude.

¶ North Atlantic fish oil, mainly capelin.

**Table 2.** Fatty acid composition (% of total) in the experimental diets

	2 g/kg EPA + DHA	10 g/kg EPA + DHA	17 g/kg EPA + DHA
14 : 0	1·0	3·3	4·5
16 : 0	14·2	11·9	8·3
18 : 0	4·2	3·2	1·5
20 : 0	0·3	0·3	0·4
Σ SFA*	20·8	19·3	15·1
16 : 1*n*-7	2·8	4·5	5·4
18 : 1*n*-7	1·2	1·6	2·2
18 : 1*n*-9	38·1	26·2	26·8
20 : 1*n*-9	0·8	7·0	10·5
22 : 1*n*-9	0·1	0·8	1·3
22 : 1*n*-11	0·4	8·1	12·5
24 : 1*n*-9	0·1	0·3	0·6
Σ MUFA†	44·1	49·8	60·8
18 : 2*n*-6	21·1	13·1	8·1
18 : 3*n*-6	0·1	0·1	0·1
20 : 2*n*-6	0·1	0·2	0·2
20 : 4*n*-6	0·3	0·2	0·2
Σ *n*-6‡	21·8	13·7	8·6
18 : 3*n*-3	11·4	11·4	4·2
20 : 4*n*-3	0·2	0·8	1·2
20 : 5*n*-3	0·4	2·4	3·6
22 : 5*n*-3	0·1	0·2	0·4
22 : 6*n*-3	0·3	1·1	2·1
Σ *n*-3	12·4	15·8	11·6
Σ PUFA§	34·2	29·5	20·1

* Also includes 12 : 0, 15 : 0, 17 : 0 and 24 : 0.

† Also includes 14 : 1*n*-5, 15 : 1, 16 : 1*n*-5, 16 : 1*n*-9, 17 : 1*n*-7, 20 : 1*n*-7 and 22 : 1*n*-7.

‡ Also includes 20 : 3*n*-6.

§ Also includes 18 : 3*n*-4.

### Weighing and sampling

Fish were weighed before they were assigned to the three dietary treatments when they were approximately 400 g, and then were distributed to the three sea cages when they were approximately 1·2 kg (body weight at the start of the experiment in sea cages) and, when the experiment finished, they had reached a slaughter weight of approximately 3·5 kg. At the end of the experiment, fifty fish from each cage were killed by an overdose of the anaesthetic metacain (MS-222; 0·08 g/l), and individual weights and lengths were recorded. Blood samples were drawn from thirty fish per cage. Erythrocytes for fatty acid analysis were collected using a Pasteur pipette after centrifugation of blood samples (2000 ***g***, 10 min, 4°C), flash frozen in liquid N_2_, and stored at −80°C. Next, the fish were gutted and scored for visceral fat content according to a scale from I to V, where I represents low levels and V the highest level. The fish were also scored for intestinal redness and swollen appearance according to a scale developed by BioMar. Samples from the muscle, liver, skin and intestine were collected and frozen at −80°C and stored for subsequent analysis of lipid composition. Samples from the mid-intestine and skin were cut into sizes suitable for histological analysis and fixed in 10 % buffered formalin. The experiment was conducted according to the National Guidelines for Animal Care and Welfare published by the Norwegian Ministry of Education and Research (Norwegian Food Safety Authority (FOTS); approval 5354). Acute handling mortalities occurred after delousing procedure at high water temperature. Veterinarians from FOTS inspected the fish, and since no obvious signs of wounds or infections were observed, the trial was allowed to continue.

### Fatty acid composition analysis

Total lipids were extracted from the blood, muscle, liver, intestine, skin and diets following the method described by Folch *et al.*^(^^17^^)^. In each dietary group, thirty fish were used for lipid analysis, and each sample included a pooled sample from two fish. One part of the chloroform–methanol phase after Folch extraction from the muscle and erythrocytes was used for the analysis of fatty acid composition of total lipids by using the method described by Mason & Waller^(^^18^^)^. Briefly, the extract was dried under N_2_ gas, and residual lipid extract was trans-methylated overnight with 2′,2′-dimethoxypropane, methanolic HCl and benzene at room temperature. The methyl esters were separated and analysed using a gas chromatograph (Hewlett Packard 6890) equipped with a split injector by using an SGE BPX70 capillary column (length, 60 m; internal diameter, 0·25 mm; and film thickness, 0·25 µm; SGE Analytical Science), flame ionisation detector and HP Chem Station software. The carrier gas was He, and the injector and detector temperatures were both 280°C. The oven temperature was increased from 50 to 180°C at the rate of 10°C/min, and then increased to 240°C at a rate of 0·7°C/min. Individual fatty acid methyl esters were identified by referring to well-characterised standards. The relative amount of each fatty acid was expressed as a percentage of the total amount of fatty acids in the analysed sample, and the absolute amount of fatty acids per g of tissue was calculated using C23 : 0 methyl ester as the internal standard.

The lipid class composition of the liver, intestine and skin was determined by evaporating a part of the Folch chloroform lipid extract under N_2_ gas and re-dissolving the residual lipid extract in hexane (Merck). PL and neutral lipids (NL) were separated using TLC by using a mixture of petroleum ether, diethyl ether and acetic acid (113:20:2, by vol.) as the mobile phase. The lipids were visualised by spraying the TLC plates with 0·2 % (w/v) 2′,7′-dichlorofluorescein in methanol, and the lipids were identified by comparing with known standards (Sigma Chemical Co.) under UV light. For the liver samples, the spots corresponding to PL and NL fractions were scraped off into glass tubes and trans-methylated following the aforementioned procedure. For the intestine and skin samples, the PL fraction was scraped off into glass tubes and dissolved in Arvidson's solution^(^^19^^)^. The PL fractions were separated using TLC by using a mobile phase composed of chloroform, methanol, acetic acid and water (100:75:6:2, by vol.). The lipids were visualised as described above, and the spots revealed were identified under UV light by comparing with known standards. The spots corresponding to phosphatidylcholine (PC), phosphatidylethanolamine (PE), phosphatidylinositol (PI) and phosphatidylserine (PS) were scraped off into glass tubes and trans-methylated following the aforementioned procedure.

### Histology

Histological analysis of the mid-intestinal tissue and skin was performed on thirty samples from each dietary group collected at the final sampling, which were fixed in 10 % phosphate-buffered formalin and stored at 4°C until analysis. The samples were dehydrated and processed according to the standard protocols. Paraplast-embedded samples were cut using a Leitz 1208 microtome (Ernst Leitz, Wetzlar GmbH) (5 µm) and stained with haematoxylin and eosin (Merck KGaA). Stained slides were examined using a standard Nikon Optiphot light microscope. Images were captured using a MicroPublisher 3.3 RTV camera and analysed using QCapture suite software (QImaging). The sections were evaluated in a blinded manner to identify any pathological or other systematic variations in tissue morphology. For the mid-intestinal tissue, variations in vacuolisation of mucosal epithelium and the presence of increased vacuolisation of supra-nuclear cytoplasm were mainly recorded. The height of mucosal folds was quantified by measuring the distance from the stratum compactum of lamina propria to the tip of the intestinal villus (in mm; Image J; National Institutes of Health). Five to ten measurements were obtained per fish, and the average of these values was used for analysis. For the skin tissue, the epidermal thickness and goblet cell number per mm length were recorded.

### Radiography

**Fig. 1.**
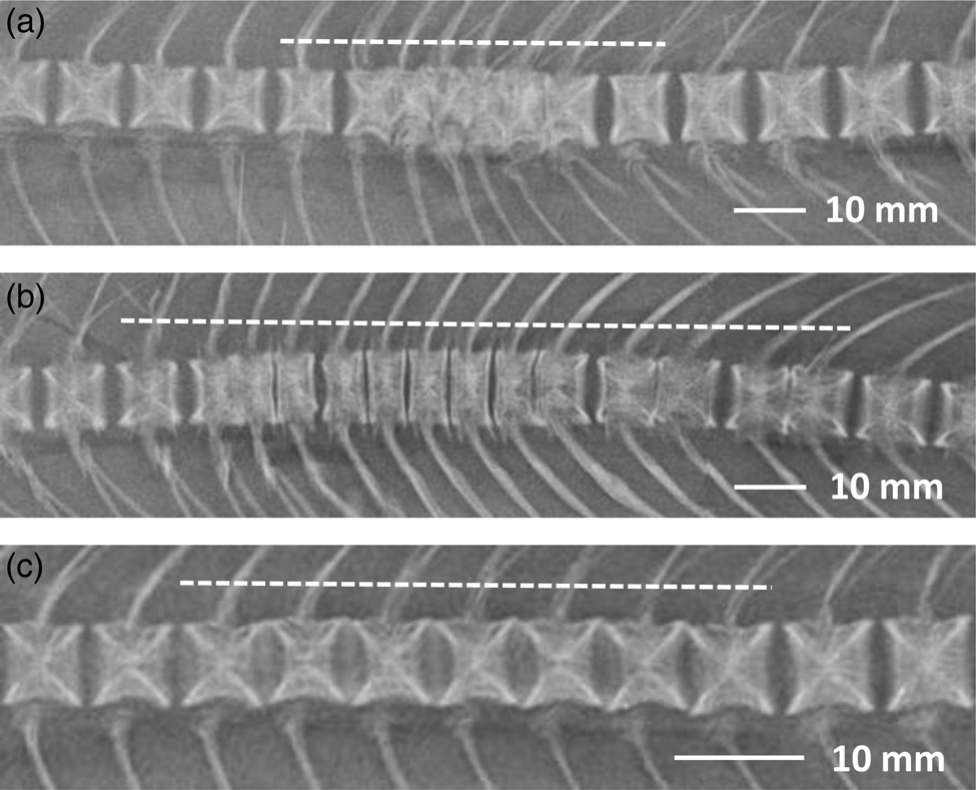
Radiography: detail of vertebral lesions. (a) Fused vertebrae, lesion located under the dorsal fin; (b) platyspondylia, located in caudal spine; (c) missing intervertebral space, lesion located in cranial spine. Dotted lines indicate the extent of the lesions.

All sampled fish at the end of the experiment were examined by radiography in order to identify any effects of diet on skeletal development and pathology. The fish were examined by X-ray at the Nofima Radiography Laboratory at Sunndalsøra, Norway. The fish were transferred to this department in the frozen form, gutted, and with one fillet removed, and were X-rayed under frozen condition. The radiography setup was semi-digital and included a standard X-ray source (Shimadzu Mobile Art) to which reusable image plates were exposed (32 kV/50 mA). The images were transferred to the computer on a plate reader (FCR Profect; Fuji Medical Inc.) and optimised before storage, i.e. automated equalisation of exposure and highlighting of edges, followed by manual adjustment of brightness and contrast (Fuji CR Console software; Fuji Medical Inc.). The digital images were evaluated visually, and variations in bone structures were recorded and classified in a blinded manner. The lesions were classified as fused vertebrae^(^^20^^)^ in various stages and to different extents, or platyspondylia (compressed vertebrae^(^^21^^)^). In addition, a different type of deviation from normal morphology was recorded, namely, groups of vertebrae missing the normal intervertebral space and having a slight vertical shift between adjacent vertebrae (Fig. 1). These deviations showed some similarities to the findings of Witten *et al.*^(^^22^^)^ who reported missing intervertebral space as a part of platyspondylia development; however, these deviations were located predominantly in the anterior spine (vertebra nos. 7 to 20). The typical location for platyspondylia development was the caudal spine. Where present, the number and location of affected vertebrae were recorded. After the variations in vertebral shape were recorded, the proportions of five vertebrae per fish were measured to quantify any such variation. Images were analysed using ImageJ software (National Institutes of Health), and vertebrae nos. 32–36, just caudal to the dorsal fin, were located. The cranio-caudal length and dorso-ventral height were measured for each of these five vertebrae, and the ratio between length and height was calculated. In this area of the spine, the normal value for length:height ratio is approximately 1. The average ratio of the five vertebrae per fish was used as a measure of vertebral proportions. These ratios were subsequently compared with visual observations on vertebral proportions. Radiography recordings are reported as percentage of fish with a particular type of lesion within a dietary group. For counts, e.g. size of lesions, values are presented as mean number of affected vertebrae±standard deviation of fish with a score of ‘1’ for affected fish. For measurements of vertebral proportions, the mean value of all fish within a dietary group±standard deviation is provided, excluding individuals with a specific lesion at the location of the measurement.

### Statistics

Statistical analyses were conducted using software JMP^®^ version 11.2.1 (SAS Institute Inc.; 1989–2007). Since the fish were pit-tagged to allow monitoring of individual fish performance, individual fish were considered as pseudo-replicates. At the end of the experiment, fifty fish from each cage were sampled and used to analyse the effects of diets on the biometric and radiographical aspects of the vertebrae (*n* 50). The effect of the three main diets on the changes in fatty acid composition of PL and NL of the muscle, liver, intestine and skin was determined for thirty fish, each sample of which was a pooled sample from two fish (*n* 15). For the histology data, individual recordings from thirty fish from each dietary group were used. Data were analysed using one-way ANOVA, and significant differences between main diets were calculated using Tukey's honestly significant difference *post hoc* test. Differences were considered significant when *P* < 0·05. Values are shown as mean values with their standard errors unless otherwise stated. The correlation between mid-intestinal macroscopy (BioMar) and microscopy (histology) scores was calculated (Pearson's correlation coefficient).

## Results

### Fish performance, biometric data and tissue lipid content

No differences were found in the mean individual start body weights of the fish assigned to one of the three experimental diets in tanks on land (*P* = 0·35). However, fish fed the 2 and 10 g/kg diets had significantly lower weight (1·1 and 1·2 kg, respectively) than those fed the 17 g/kg diet (1·3 kg; *P* < 0·0001) at the final sampling on land before transport to sea cages. However, no differences were found in the final body weight (*P* = 0·65), total length (*P* = 0·71), hepatosomatic index (*P* = 0·19) or cardiosomatic index (*P* = 0·10) (Table 3) among the three dietary groups at the final sampling in sea cages. Nevertheless, fish fed the 2 g/kg diet showed the lowest weight (3·3 kg) compared with those fed the 10 and 17 g/kg diets (3·5 kg).

**Table 3.** Performance and tissue lipid content in Atlantic salmon (*Salmo salar*) fed three different dietary levels (2, 10 or 17 g/kg) of EPA + DHA from 400 g to slaughter size (Mean values with their standard errors using individual fish as the statistical unit (*n* 50))

	2 g/kg EPA + DHA		10 g/kg EPA + DHA		17 g/kg EPA + DHA		
	Mean	sem	Mean	sem	Mean	sem	ANOVA: *P*
IBW (g) – start tanks on land	369	12	356	14	341	14	0·35
BW (g) – start sea cages	1095^b^	26	1183^b^	31	1326^a^	43	<0·0001
FBW (g)	3346	80	3526	158	3515	200	0·65
Length (cm)	612	4·5	620	9·0	621	10·7	0·71
HSI (%)	1·27	0·02	1·30	0·04	1·23	0·03	0·19
CSI (%)	0·11	0·001	0·11	0·002	0·11	0·002	0·10
Liver fat (%)	9·1^a^	0·72	6·8^b^	0·51	7·6^a,b^	0·37	0·02
Muscle fat (%)*	15·0^a^	0·40	16·4^a,b^	0·56	17·9^b^	0·50	0·0006
Intestine fat (%)*	15·0^a^	1·65	8·8^b^	0·64	11·0^a,b^	1·50	0·01
Visceral fat score	3·3^a^	0·2	2·6^b^	0·2	2·9^a,b^	0·2	0·01

IBW, initial body weight; BW, body weight; FBW, final body weight; HSI, hepatosomatic index; CSI, cardiosomatic index.

^a,b^ Mean values within a row with unlike superscript letters were significantly different (*P* < 0·05; one-way ANOVA followed by Tukey's honestly significant difference test).

* For muscle and intestine fat content, thirty fish were used.

Salmon fed the 2 g/kg EPA + DHA diet during 13 months showed significantly higher levels of fat in the liver (*P* = 0·02) and intestine (*P* = 0·01) than those fed the 10 g/kg diet and a significant lower muscle fat content (*P* = 0·0006) than that in the fish fed the 17 g/kg diet. In addition, fish fed the 2 g/kg diet showed significantly more fat deposition around the viscera than those fed the other two diets (*P* = 0·01).

High mortalities were observed in the fish fed 2 and 10 g/kg diets after the delousing processes; in particular, these values were high in July and August, when the water temperature was relatively high. The accumulated mortality was 63, 52 and 16 % for the fish fed the 2, 10 and 17 g/kg diet, respectively. Veterinarians subjected the dead fish to medical evaluation, and the fish were further screened for salmonid alphavirus and piscine retrovirus and the results were negative for both. No signs of any known disease were found, and the fish were concluded to have probably died due to the handling stress after delousing at high water temperatures.

### Muscle fatty acid composition

The fatty acid composition of the muscle largely reflected the fatty acid composition of the diets (Table 4). After the fish were fed the experimental diets for 13 months, a gradual decrease in the content of EPA and DHA in the fillet was observed with decreasing dietary levels of these fatty acids. The EPA and DHA content in fish fillet was 3·1, 5·3 and 7·1 mg/g in fish fed the 2, 10 and 17 g/kg diet, respectively. Despite this, the level of *n*-3 PUFA was significantly higher in the muscle of the fish fed the 10 g/kg diet than in that of the fish fed the 2 and 17 g/kg diets, owing to the higher dietary levels of 18 : 3*n*-3 in the 2 and 10 g/kg diets. Similarly, the fish fed the 17 g/kg diet had higher levels of 20 : 1*n*-9 and 22 : 1*n*-11, and thus higher levels of total monoenes, reflecting the dietary fatty acid profile. The total *n*-6 PUFA content in the muscle was gradually increased when the dietary EPA and DHA content decreased. Thus, the *n*-6:*n*-3 ratio was significantly increased in the muscle of the fish fed the 2 g/kg diet (1·8 (sem 0·03)) compared with that in the fish fed the 10 and 17 g/kg diets (1·1 (sem 0·01) for both the dietary groups).

**Table 4.** Fatty acid composition (mg/g) in the fillet of Atlantic salmon (*Salmo salar*) fed different levels of EPA and DHA for 13 months (Mean values with their standard errors; thirty fish are behind the analysis in each dietary group, being each sample originated from a pooled sample from two fish (*n* 15))

	2 g/kg EPA + DHA		10 g/kg EPA + DHA		17 g/kg EPA + DHA	
	Mean	sem	Mean	sem	Mean	sem
14 : 0	1·3^c^	0·09	3·1^b^	0·11	5·0^a^	0·17
16 : 0	13·5	0·45	14·2	0·45	13·1	0·40
18 : 0	4·5^a^	0·15	4·1^a^	0·12	2·9^b^	0·08
Σ SFA*	19·8	0·66	22·0	0·70	21·7	0·66
16 : 1*n*-7	3·0^c^	0·12	4·9^b^	0·17	6·5^a^	0·21
18 : 1*n*-7	1·9^c^	0·10	2·6^b^	0·11	3·4^a^	0·08
18 : 1*n*-9	40·2	1·29	36·2	1·03	39·1	1·24
20 : 1*n*-9	2·1^c^	0·20	6·7^b^	0·24	12·4^a^	0·48
22 : 1*n*-11	0·9^c^	0·21	6·0^b^	0·25	12·5^a^	0·64
Σ MUFA†	51·7^c^	1·69	61·6^b^	1·86	79·5^a^	2·57
18 : 2*n*-6	19·0^c^	0·62	15·8^b^	0·40	12·8^a^	0·38
20 : 3*n*-6	0·8^a^	0·06	0·6^b^	0·04	0·5^b^	0·01
20 : 4*n*-6	0·5^a^	0·01	0·4^b^	0·02	0·3^c^	0·01
Σ *n*-6‡	22·2^a^	0·73	18·4^b^	0·48	15·1^c^	0·44
18 : 3*n*-3	8·7^b^	0·28	9·8^a^	0·28	4·6^c^	0·16
20 : 5*n*-3	1·5^c^	0·07	2·5^b^	0·12	3·2^a^	0·19
22 : 5*n*-3	0·5^c^	0·02	0·8^b^	0·04	1·3^a^	0·04
22 : 6*n*-3	1·6^c^	0·09	2·8^b^	0·08	3·9^a^	0·08
EPA + DHA	3·1^c^	0·14	5·3^b^	0·16	7·1^a^	0·23
Σ *n*-3§	12·7^b^	0·41	16·7^a^	0·41	13·6^b^	0·39
*n*-6:*n*-3	1·8^a^	0·03	1·1^b^	0·01	1·1^b^	0·01

^a,b,c^ Mean values within a row with unlike superscript letters were significantly different (*P* < 0·05; one-way ANOVA followed by Tukey's honestly significant difference test).

* Also includes 15 : 0, 17 : 0, 20 : 0 and 24 : 0.

† Also includes 14 : 1*n*-5, 16 : 1*n*-5, 16 : 1*n*-9, 17 : 1*n*-7, 20 : 1*n*-7, 20 : 1*n*-11, 22 : 1 *n*-7 and 24 : 1*n*-9.

‡ Also includes 18 : 3*n*-6 and 20 : 2*n*-6.

§ Also includes 20 : 3*n*-3.

### Liver fatty acid composition

Fish fed the 2 g/kg diet had significantly higher amount of NL in the liver (23·6 (sem 3·4) mg/g; *P* = 0·009) than that in the fish fed the 10 and 17 g/kg diets (14·0 (sem 2·2) and 13·3 (sem 1·3) mg/g, respectively; Table 5). The fatty acid compositions of liver PL and NL were affected by the fatty acid composition of the diets (Table 5). The decreasing dietary levels of EPA and DHA led to a significant increase in the percentage of 18 : 1*n*-9, 18 : 2*n*-6, 20 : 3*n*-6 and 20 : 4*n*-6 in the PL and NL fractions. The increase in 18 : 1*n*-9 and 18 : 2*n*-6 was primarily caused by the increasing dietary content of these fatty acids, and the increase in the elongation and desaturation products of 18 : 2*n*-6, 20 : 3*n*-6 and 20 : 4*n*-6 was particularly relevant in the PL fraction. In addition, decrease in the dietary levels of EPA and DHA led to a gradual decrease in the amount of *n*-3 VLC-PUFA in both PL and NL. Interestingly, the EPA and DHA content in the NL fraction from fish fed the 10 and 17 g/kg diets did not differ markedly. In contrast the EPA and particularly DHA content in the NL fraction from the fish fed the 2 g/kg diet was nearly depleted, whereas in the PL fraction the content was halved compared with the levels present in the 17 g/kg dietary group.

**Table 5.** Fatty acid composition (% of total) in the liver of Atlantic salmon (*Salmo salar*) fed different levels of EPA and DHA for 13 months (Mean values with their standard errors; thirty fish are behind the analysis in each dietary group, being each sample originated from a pooled sample from two fish (*n* 15))

	Liver PL						Liver NL					
	2 g/kg EPA + DHA		10 g/kg EPA + DHA		17 g/kg EPA + DHA		2 g/kg EPA + DHA		10 g/kg EPA + DHA		17 g/kg EPA + DHA	
	Mean	sem	Mean	sem	Mean	sem	Mean	sem	Mean	sem	Mean	sem
16 : 0	10·7^b^	0·33	12·8^a^	0·56	12·2^a^	0·32	7·9^b^	0·30	10·2^a^	0·40	9·9^a^	0·27
18 : 0	6·7^a^	0·13	6·2^a^	0·19	4·5^b^	0·12	6·4^a^	0·21	4·8^b^	0·15	4·1^b^	0·20
Σ SFA*	18·2^b^	0·41	20·2^a^	0·67	18·7^a,b^	0·38	16·1^b^	0·46	17·9^b^	0·52	17·6^a,b^	0·46
16 : 1*n*-7	1·5^b^	0·12	1·9^b^	0·16	2·4^a^	0·10	3·1^c^	0·15	4·3^b^	0·22	5·0^a^	0·10
18 : 1*n*-9	29·2^a^	0·79	21·7^b^	0·99	20·6^b^	0·52	41·4^a^	1·45	31·7^b^	1·07	28·8^b^	0·75
20 : 1*n*-9	1·8^c^	0·06	3·8^b^	0·22	5·8^a^	0·20	3·2^c^	0·12	6·1^b^	0·28	8·6^a^	0·31
Σ MUFA†	34·9^a^	0·92	30·4^b^	1·26	32·8^a,b^	0·80	52·0	1·39	48·1	1·50	51·4	1·08
18 : 2*n*-6	11·9^a^	0·26	7·5^b^	0·15	5·3^c^	0·12	14·1^a^	0·37	10·1^b^	0·16	7·2^c^	0·28
20 : 3*n*-6	3·4^a^	0·14	1·5^b^	0·05	0·8^c^	0·04	1·7^a^	0·12	0·8^b^	0·04	0·6^b^	0·07
20 : 4*n*-6	5·0^a^	0·14	3·1^b^	0·14	1·5^c^	0·05	1·0	0·18	0·7	0·12	0·6	0·09
Σ *n*-6‡	22·3^a^	0·36	13·7^b^	0·15	8·8^c^	0·15	19·3^a^	0·41	13·4^b^	0·24	10·0^c^	0·26
18 : 3*n*-3	3·2^a^	0·13	2·8^b^	0·14	1·1^c^	0·04	4·8^b^	0·16	5·7^a^	0·16	2·5^c^	0·08
20 : 4*n*-3	0·1^c^	0·01	0·6^b^	0·04	0·7^a^	0·02	0·2^c^	0·05	1·2^b^	0·07	1·5^a^	0·06
20 : 5*n*-3	6·3^c^	0·21	9·1^b^	0·36	10·6^a^	0·22	1·8^b^	0·25	4·0^a^	0·46	3·9^a^	0·29
22 : 5*n*-3	2·3^c^	0·10	2·8^b^	0·10	3·1^a^	0·04	0·4^b^	0·06	0·8^a^	0·12	0·9^a^	0·11
22 : 6*n*-3	8·9^c^	0·34	16·0^b^	0·56	18·9^a^	0·38	1·0^b^	0·18	3·4^a^	0·72	3·1^a^	0·35
Σ *n*-3§	21·2^c^	0·48	31·7^b^	0·67	34·7^a^	0·50	8·6^c^	0·56	15·7^a^	1·14	12·3^b^	0·66
*n*-6:*n*-3	1·06^a^	0·03	0·43^b^	0·01	0·25^c^	0·01	2·32^a^	0·10	0·90^b^	0·05	0·83^b^	0·03
Σ Fatty acids (mg/g)	15·3	0·8	14·0	1·0	14·2	0·7	23·6^a^	3·4	14·0^b^	2·2	13·3^b^	1·3

PL, phospholipids; NL, neutral lipids.

^a,b,c^ Mean values within a row with unlike superscript letters were significantly different (*P* < 0·05; one-way ANOVA followed by Tukey's honestly significant difference test).

* Also includes 14 : 0, 15 : 0, 17 : 0, 20 : 0 and 22 : 0.

† Also includes 14 : 1*n*-5, 15 : 1, 16 : 1*n*-9, 17 : 1*n*-7, 18 : 1*n*-7, 18 : 1*n*-11 and 24 : 1*n*-9.

‡ Also includes 18 : 3*n*-6 and 20 : 2*n*-6.

§ Also includes 18 : 3*n*-3 and 20 : 3*n*-3.

### Erythrocyte fatty acid composition

Dietary reductions in EFA had a clear effect on the fatty acid composition of erythrocytes (Table 6). The percentage of monoenes in the erythrocytes did not differ among the fish fed the experimental diets. This was explained by the gradual increase and decrease in 18 : 1*n*-9 and 20 : 1*n*-9 in the cells, respectively, as dietary EPA and DHA were reduced. Erythrocytes from salmon fed the 2 g/kg diet had marked reduction in EPA and DHA, whereas the amount of 18 : 3*n*-3 and, to a lesser extent 20 : 3*n*-3, were increased compared with the erythrocytes of fish fed the 10 and 17 g/kg diets. Nevertheless, unlike the liver, the erythrocytes could selectively conserve higher relative levels of EFA. The gradual decrease in *n*-3 fatty acids when the fish were fed decreasing levels of EPA and DHA was accompanied by an increase in *n*-6 fatty acids, particularly 18 : 2*n*-6, 20 : 3*n*-6 and 20 : 4*n*-6. The relative increase in *n*-6 fatty acids was very similar to that observed in the PL fraction of the liver. However, since the total *n*-3 PUFA levels were more conserved in erythrocytes, the *n*-6:*n*-3 ratio was not as high in the fish fed the 2 g/kg diet as it was in the liver.

**Table 6.** Fatty acid composition (% of total) in the erythrocytes of Atlantic salmon (*Salmo salar*) fed different levels of EPA and DHA for 13 months (Mean values with their standard errors; thirty fish are behind the analysis in each dietary group, being each sample originated from a pooled sample from two fish (*n* 15))

	2 g/kg EPA + DHA		10 g/kg EPA + DHA		17 g/kg EPA + DHA	
	Mean	sem	Mean	sem	Mean	sem
16 : 0	16·2^b^	0·19	17·1^a^	0·12	16·4^b^	0·15
18 : 0	7·5^a^	0·19	6·5^b^	0·23	5·7^c^	0·18
Σ SFA*	24·5	0·23	25·0	0·27	24·5	0·33
18 : 1*n*-7	1·4^c^	0·01	1·6^b^	0·04	1·9^a^	0·04
18 : 1*n*-9	13·7^a^	0·35	11·2^b^	0·44	8·9^c^	0·37
20 : 1*n*-9	1·1^c^	0·03	2·5^b^	0·21	3·8^a^	0·11
Σ MUFA†	17·3	0·50	17·6	0·68	17·8	0·46
18 : 2*n*-6	10·2^a^	0·29	6·4^b^	0·15	4·0^c^	0·13
18 : 3*n*-6	0·4^a^	0·03	0·1^b^	0·02	0·1^b^	0·05
20 : 2*n*-6	1·4^a^	0·04	1·0^b^	0·04	0·8^c^	0·02
20 : 3*n*-6	3·4^a^	0·08	1·2^b^	0·03	0·6^c^	0·02
20 : 4*n*-6	5·4^a^	0·14	3·0^b^	0·12	1·9^c^	0·09
Σ *n*-6	20·8^a^	0·34	11·6^b^	0·12	7·3^c^	0·18
18 : 3*n*-3	3·1^a^	0·09	2·6^b^	0·21	1·0^c^	0·04
20 : 3*n*-3	0·8^a^	0·04	0·6^b^	0·02	0·2^c^	0·02
20 : 5*n*-3	9·6^c^	0·23	12·4^b^	0·39	14·1^a^	0·31
22 : 5*n*-3	3·7	0·10	3·5	0·11	3·8	0·10
22 : 6*n*-3	16·4^c^	0·54	22·7^b^	0·77	27·6^a^	0·36
Σ *n*-3‡	33·7^c^	0·72	42·2^b^	0·73	47·1^a^	0·58
*n*-6:*n*-3	0·62^a^	0·02	0·28^b^	0·01	0·16^c^	0·00

^a,b,c^ Mean values within a row with unlike superscript letters were significantly different (*P* < 0·05; one-way ANOVA followed by Tukey's honestly significant difference test).

* Also includes 12 : 0, 14 : 0 and 17 : 0.

† Also includes 16 : 1*n*-7, 18 : 1*n*-11 and 24 : 1*n*-9.

‡ Also includes 20 : 4*n*-3.

### Membrane composition of the intestine and skin

The effect of the diets on the fatty acid composition of the main PL classes of the intestinal and skin membranes was analysed. Regardless of the diet and tissue, each of the different PL classes was characterised by a specific compositional pattern of fatty acids. Thus, the PC fraction from both the tissues was characterised by the highest relative levels of 16 : 0, 18 : 1*n*-9 and EPA. The PS fraction contained the highest proportion of 18 : 0, 22 : 5*n*-3 and DHA in both the tissues, and the relative level of ARA was also the highest in the skin. ARA was also highly present in the PI fraction of the skin and intestine. The PE fraction for both the tissues was characterised by high percentages of DHA and 18 : 2*n*-6. This last fatty acid was also present in the PC fraction in relatively high percentages.

**Fig. 2.**
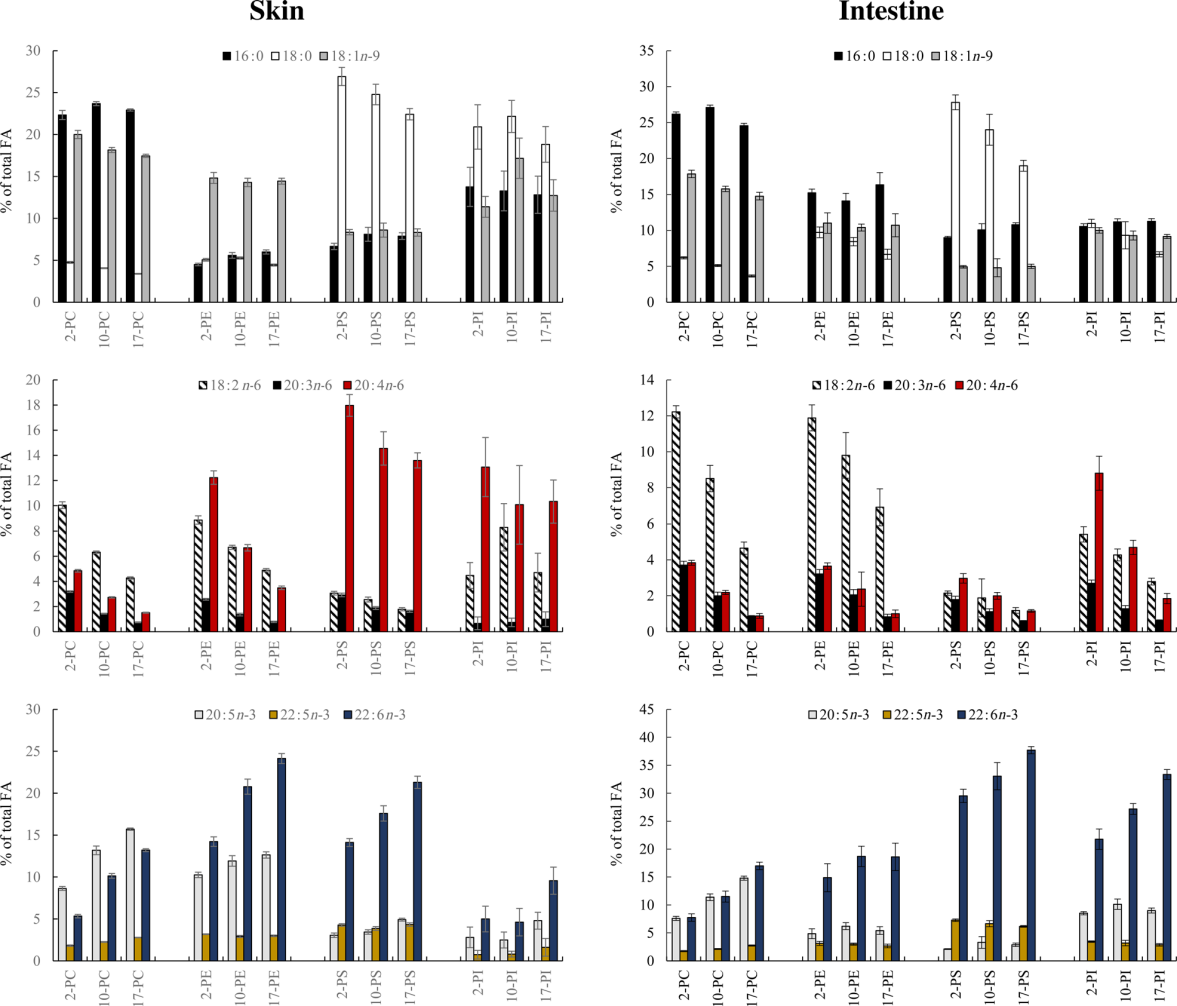
Fatty acids (FA; measured as the percentage of total FA) in the intestine and skin phospholipid classes phosphatidylcholine (PC), phosphatidylethanolamine (PE), phosphatidylserine (PS) and phosphatidylinositol (PI) of salmon fed three different dietary levels: 2, 10 or 17 g/kg EPA + DHA from 400 g to slaughter size. Data are means, with standard errors represented by vertical bars. A total of thirty fish are behind the analysis in each dietary group, being each sample originated from a pooled sample from two fish (*n* 15).

Dietary effects were evident on the relative distribution of the characteristic above-mentioned fatty acids in the main PL classes analysed (Fig. 2 and Supplementary Table S2). PC was the most influenced PL class, in which all the fatty acids shown in Fig. 2 were significantly affected by the diet in both the skin and intestine. The tissue content of EPA and DHA in PC was reduced by half in the fish fed the 2 g/kg diet compared with that from fish fed the 17 g/kg diet. This was compensated by a moderate increase in 18 : 0 and 18 : 1*n*-9, whereas the levels of total *n*-6 PUFA almost tripled. In particular, 20 : 3*n*-6 was increased by 4·3 times in the fish fed the 2 g/kg diet in both the tissues, and 20 : 4*n*-6 was increased by 3·2 and 4·4 times in the skin and intestine, respectively.

In the PE fraction of the skin, all the fatty acids shown in Fig. 2 were significantly affected by the dietary levels of EPA and DHA with the exception of 18 : 1*n*-9 and 22 : 5*n*-3. The *n*-3 fatty acids and *n*-6 fatty acids were gradually decreased and increased, respectively, as the fish were fed decreasing levels of EPA and DHA. In particular, DHA decreased by 10 percentage points in the skin PE fraction between the fish fed the 17 g/kg diet and those fed the 2 g/kg diet; this was compensated by a 9 percentage points increase in ARA. Changes in the intestinal PE fraction were more moderate, and only the *n*-6 fatty acids and 18 : 0 were significantly increased by dietary EPA and DHA reductions, whereas the *n*-3 fatty acids remained at steady levels.

In the skin, PI was the most conserved fraction, with no significant differences in any of the fatty acids shown in Fig. 2, and only DHA showed a tendency (*P* = 0·07) to increase in the fish fed the 17 g/kg diet. In contrast, in the intestine, the fatty acid composition of the PI fraction was to a large extent influenced by the dietary EPA and DHA content. Only 16 : 0, 18 : 1*n*-9, and 22 : 5*n*-3 remained stable, whereas the levels of the *n*-6 fatty acids and *n*-3 fatty acids increased and decreased, respectively, as the fish were fed lower levels of EPA and DHA. These changes were primarily attributed to a marked decrease in DHA and a concomitant increase in ARA, which was almost five times in the fish fed the 2 g/kg diet compared with those receiving the 17 g/kg diet.

Dietary effects on the fatty acid composition of the PS fraction were very similar in both the intestine and skin. Both showed a gradual increase in 18 : 0 and *n*-6 fatty acids and a concomitant decrease in DHA as the levels of EPA and DHA decreased. Furthermore, the levels of EPA were also significantly decreased in the skin PS fraction of the fish fed the 10 and 2 g/kg diets. In the intestine PS fraction, the levels of 22 : 5*n*-3 were significantly increased by decreasing dietary levels of EPA and DHA, suggesting that 22 : 5*n*-3, to a certain extent, replaces DHA in this fraction when the DHA content is reduced.

### Morphology of the intestine and skin

**Fig. 3.**
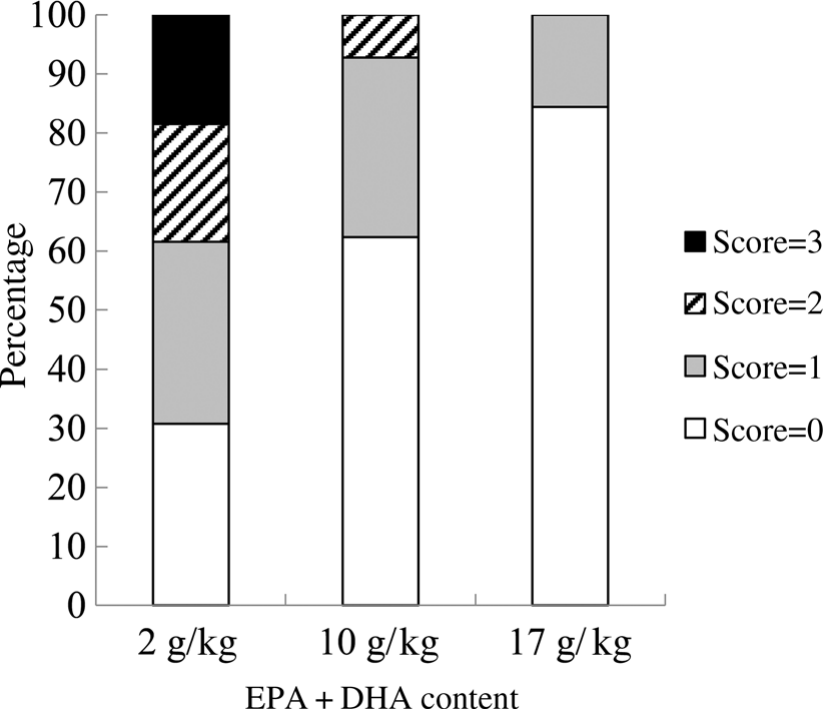
Intestinal health score based on the method developed by BioMar. All sampled fish were evaluated (*n* 50).

At the end of the experiment, the intestinal redness and swollen appearance of all the fish were visually evaluated according to a scoring method developed by BioMar ranging in a scale from 0 to 3, in which 0 represents no signs of redness or swollen intestine and three indicates severe redness and swollen intestine. Approximately 20 % of the fish fed the 2 g/kg diet scored 3; 20 % scored 2; and only 30 % scored 0. Only 10 % of the fish fed the 10 g/kg diet showed a moderate degree of intestinal alterations (score 2), whereas 60 % of the fish scored 0. The majority of the fish fed the 17 g/kg diet (80 %) scored 0, and the remaining scored 1 (Fig. 3).

**Fig. 4.**
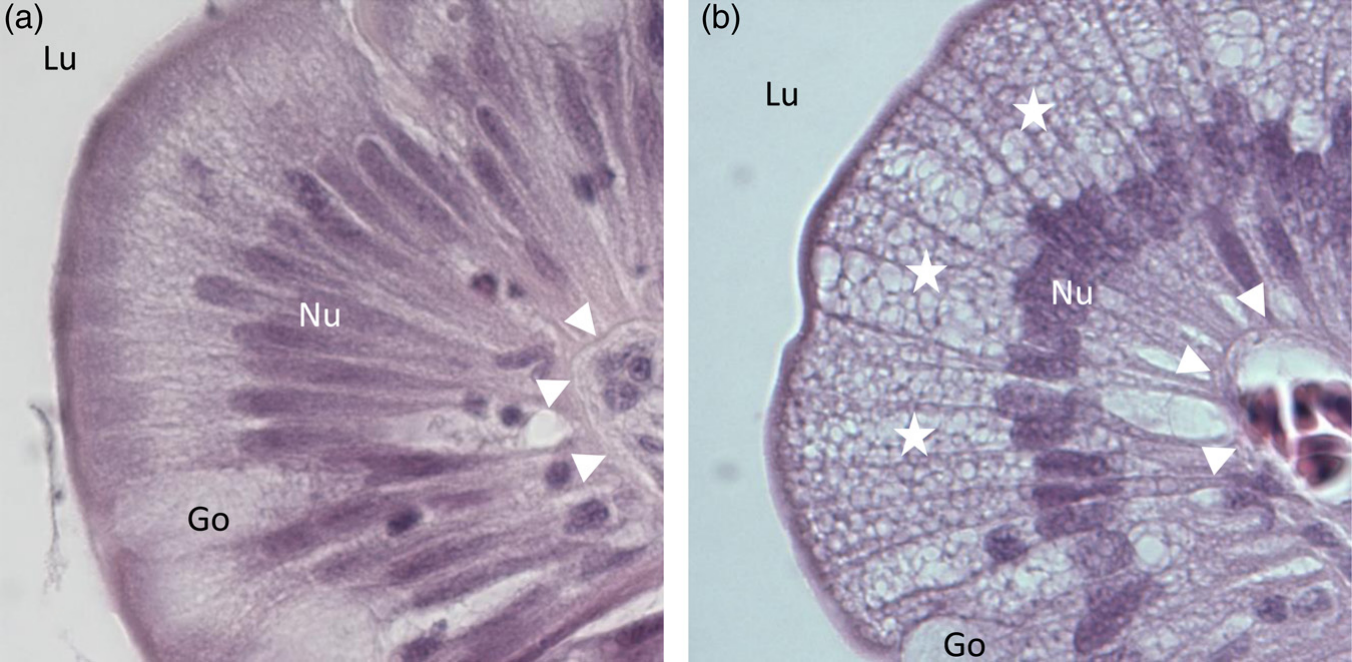
Histology of mid-intestinal mucosa in Atlantic salmon (*Salmo salar*). (a) Normal mucosa, tip of mucosal fold. (b) Mucosa with increased vacuolisation in supranuclear region of enterocytes (stars). Lu, intestinal lumen; Nu, nuclei of enterocytes; Go, goblet cells (mucus production); arrowheads, basal lamina. Haematoxylin and eosin, 600×.

Microscopic analysis of the mid-intestinal samples showed no specific intestinal pathology, in particular, no signs of inflammation in the form of increased cell infiltration, hyperaemia, or disturbed tissue integrity. Some sections showed increased vacuolisation of enterocytes, in particular in the supra-nuclear cytoplasm. The incidence of abnormal vacuolisation was significantly different among the fish fed the different diets, occurring only in the two groups fed the lowest dietary levels of EPA and DHA (Fig. 4). Thus, 50, 25 and 0 % of the fish fed 2, 10 and 17 g/kg EPA + DHA, respectively, had an increased vacuolisation of enterocytes. When comparing histology scores for vacuolisation with the macroscopic score for swollen and red intestines, a correlation of 0·60 was found (Pearson's correlation coefficient, *P* < 0·0001). By analysing correlation between these parameters within each of the diets, the correlation coefficients were 0·62 (*P* < 0·0001), 0·57 (*P* < 0·001) and not significant, for the 2, 10 and 17 g/kg dietary groups, respectively. However, the average heights of mucosal folds did not differ significantly between the dietary treatments and were 1·34 (sem 0·03), 1·29 (sem 0·04) and 1·34 (sem 0·04) mm for diets with 2, 10 and 17 g/kg EPA + DHA, respectively. No significant effects on the epidermis thickness and goblet cell number were noted in the fish fed the different experimental diets (data not shown).

### Radiography analysis of the spine

Radiography analysis showed many fish having specific pathological lesions in the spine, or some more subtle deviations of unknown aetiology and importance. The number of fish without any lesions differed significantly between the diets, with a higher number of normal fish found in the 17 g/kg dietary group (56 %) than in the remaining two dietary groups (38 and 35 %, respectively). The affected vertebrae showed no systematic deviation in shape.

Specific lesions, i.e. fusions and platyspondylia, were recorded in 18 % of fish (Table 7). No significant differences were found among the diet groups, either in percentage of affected fish or in the extent of lesions, despite some variation in the observed values. Conversely, the incidence of fish having missing intervertebral space varied significantly with diet. This type of deviation was found in 30 % of fish of the 2 and 10 g/kg dietary groups compared with 7 % in the 17 g/kg dietary group. However, the sizes of the lesions were not different among the dietary groups.

**Table 7.** Radiography of fish at the end of the experiment* (Mean values with their standard errors and percentages; *n* 50)

	2 g/kg EPA + DHA	10 g/kg EPA + DHA	17 g/kg EPA + DHA	Average mean
No remark (%)	38^a^	35^a^	56^b^	45
Fusions (%)	9·2	11·6	16·5	12·9
Average size of lesion				
Mean	3·0	3·0	4·9	4·0
sem	1·7	1·5	3·3	
Platyspondylia (%)	4·6	8·7	3·3	5·3
Average size of lesion				
Mean	25	8·8	21	16
sem	9·2	5·7	9·2	
Missing intervertebral space (%)	30·8^a^	30·4^a^	6·6^b^	20·9
Average size of lesion				
Mean	4·1	3·9	4·7	4·1
sem	1·9	1·6	1·2	
Length:height ratio of vertebrae				
Mean	0·95^a^	0·96^a,b^	0·97^b^	0·96
sem	0·035	0·042	0·035	
Reduced length:height ratio (%)	27	27	22	25

^a,b^ Mean values within a row with unlike superscript letters were significantly different (*P* < 0·05; one-way ANOVA followed by Tukey's honestly significant difference test).

* Classification of lesions into main groups (presented as percentage of fish in group) and quantification of size of lesion (no. of affected vertebrae per fish with this type of lesion). Length:height ratio was measured in five vertebrae per fish (vertebrae no. 32–36).

**Fig. 5.**
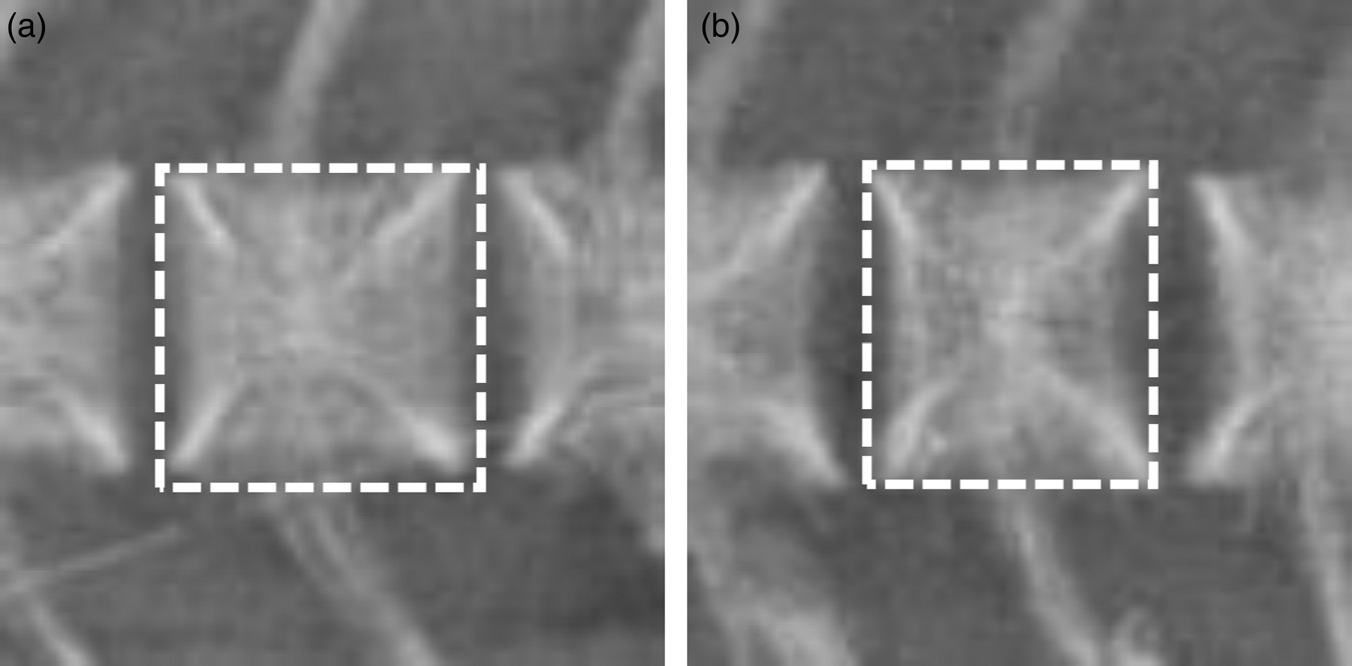
Radiography: detail of vertebrae with different length:height ratios. (a) Vertebra with normal proportions, length:height ratio about 1; (b) vertebra with reduced length, length:height ratio 0·92. Outlines of structures are indicated by dotted lines.

The measured length:height ratios of vertebrae (Fig. 5) showed a significant relation with dietary EPA and DHA contents, showing relatively shorter vertebrae (lower length:height ratio) with lower dietary content of EPA and DHA (Table 7). The differences in average values were small (between 0·95 and 0·97), and the variation within each of the groups was high (between 0·85 and 1·05) for individual fish. The measured ratios were compared with the images, and a subjective marginal value of 0·93 was set. The length:height ratio of <0·93 was found to be associated with the visual shortening of vertebrae, and the fish were recorded to have reduced vertebral length. The percentage of fish with visibly reduced vertebral length was higher in the 2 and 10 g/kg groups (27 %) than in the 17 g/kg group (22 %), although no significant difference was found.

## Discussion

Changes in aquafeed formulation have necessitated the reassessment of the nutritional requirements for *n*-3 VLC-PUFA in Atlantic salmon^(^^2^^)^. Therefore, in this study, the effects of fish diet containing three levels of EPA and DHA (0·7, 3·5 and 5·7 % of total fatty acids corresponding to 2, 10 and 17 g/kg feed) on fish performance and health condition in sea cages were evaluated. The 17 g/kg feed diet had a composition close to that of a diet used currently in Norwegian commercial salmon farming.

No significant differences in final weights were found among the fish in the different dietary groups in our study, which was contradictory to the findings of most other studies showing that low dietary levels of EPA and DHA reduced growth^(^^13^^,^^23^^)^. However, in the present study, fish were fed the same three experimental diets in tanks on land from 400 g to 1·2 kg before they were transferred to sea cages and, during this period, the fish fed the two lowest dietary levels of EPA and DHA showed significantly lower growth rates. During the time in sea cages, the fish with the lowest weight at the starting point (2 g/kg dietary group) seemed to show compensatory growth, because of which the final body weight remained approximately the same in all the dietary groups at the final sampling. However, the fish were subjected to repeated handling stress during transport from one location to another and several rounds of delousing coinciding with high water temperatures. These challenging environmental conditions might have been responsible for the lower growth rate observed in the present trial, unlike that found in other studies^(^^13^^,^^15^^)^. Nonetheless, the possibility that the final weights would be different between the dietary groups if the fish would have had better growth performance cannot be excluded.

The EFA requirements may also be determined based on fish health and welfare. Mortalities in salmon fed the 2 and 10 g/kg diets (63 and 52 %, respectively) were higher than those in the 17 g/kg dietary group (16 %). The majority of mortalities occurred during the initial days after treatment against sea lice at high water temperature; therefore, the mortalities could be assumed to be caused by handling stress, and the fish fed the lowest dietary *n*-3 levels could be considered to be less robust to handle stress than those fed higher dietary levels of these fatty acids. This finding is in agreement with that of a previous study in which fish fed a vegetable oil (VO)-based diet with approximately 10 g/kg feed of EPA and DHA experienced transportation-induced stress, leading to 30 % mortality^(^^24^^)^. The lack of major differences in the final body weight between the different dietary groups might partly be attributed to the individual differences among fish within each cage, where the survivors were the high performers that could better utilise the diet resources despite the low dietary EPA and DHA levels compared with the fish that died. The mortality within each cage was unrelated to the individual pre-dietary history of fish from 40 g to 400 g, suggesting that the feed consumed by fish from 400 g until slaughter size was what influenced the mortality in this trial.

The lower growth of fish fed the 2 and 10 g/kg diets observed in the first period in land tanks together with the high accumulated mortality indicate that a dietary content of 10 g/kg EPA and DHA is not sufficient to sustain growth and fish robustness under demanding environmental conditions. This is in contradiction to a recent long-term feeding trial showing that Atlantic salmon fed 1·4 % EPA and DHA of total fatty acids (corresponding to 4 g/kg feed) experienced no mortalities^(^^13^^)^. However, that study was performed under controlled environmental conditions in tanks on land, which might explain the discrepancy between the mortality rates since, in this study, the fish were subjected to major environmental challenges during the sea-cage period. Another study showed that Atlantic salmon reared in sea water through an entire production cycle under commercial conditions and fed two different levels of EPA and DHA (5 and 8 % of total fatty acids corresponding to 16 and 26 g/kg feed) showed mortalities in the range of those commonly observed under commercial settings, although the fish experienced diverse environmental challenges, including lice, pancreas disease outbreak and gill infections^(^^15^^)^. However, the lowest EPA and DHA content in the feed in that trial was similar to that in the higher dose tested in the present study, which is representative of the commercial levels used at present. This dietary group also showed the expected 16 % mortality that is normally found in sea cages^(^^14^^)^. Taken together, the findings of these studies indicate that 17 g/kg dietary levels of EPA and DHA do not negatively influence fish health and performance under commercial settings^(^^14^^,^^15^^)^.

In agreement with previously reported findings^(^^1^^)^, the content of EPA and DHA in the fillet was reduced by reduced dietary levels of these fatty acids. However, although the dietary level of EPA and DHA was 8·5 times lower in the 2 g/kg diet than in the 17 g/kg diet, the muscle fillet level of the EFA was only reduced by 2·3 times. Further, although the amount of the dietary precursor 18 : 3*n*-3 was the same in the 2 and 10 g/kg diets, 18 : 3*n*-3 muscle content was significantly reduced in fish fed the 2 g/kg diet, indicating that this fatty acid was elongated and desaturated to its longer-chain homologues to a higher extent than in fish fed the 10 g/kg diet, probably to compensate for the lack of dietary EPA and DHA. This indicates that salmon fed a diet that almost lacks *n*-3 VLC-PUFA show a considerable endogenous production of these fatty acids. This is in agreement with the findings of other studies showing that the lack of *n*-3 VLC-PUFA stimulates the *n*-3 fatty acid biosynthetic pathway in Atlantic salmon^(^^25^^)^. Atlantic salmon is an important contributor to marine lipids in humans; hence, determining whether salmon fed only 2 g/kg of EPA and DHA during the entire production cycle still contain a considerable amount of these fatty acids owing to its innate fatty acid production capacity is interesting. The International Society for the Study of Fatty Acids and Lipids recommends a weekly intake of 3·5 g of EPA and DHA for humans. Therefore, a serving portion of 200 g from fish fed the 2, 10 and 17 g/kg diets would provide 17·7, 30·2 and 40·6 % of the weekly recommended intake, respectively.

The significant decrease in muscle fat deposition observed in the fish fed the 2 g/kg diet, together with the significant increase in fat deposition in the liver, intestine and around the viscera compared with that observed in fish fed the 10 g/kg diet, suggests not only an inefficient way of utilising dietary energy but also, more importantly, a failure in the mechanisms regulating lipid deposition and mobilisation, which might compromise fish welfare. In mammals, an imbalance in fat deposition/mobilisation can lead to developmental metabolic disorders such diabetes, non-alcoholic fatty liver disease and dyslipidaemia^(^^26^^)^. Further, enhanced fat accumulation in the liver could be associated with metabolic risk factors for the metabolic syndrome and could increase the risk of CVD^(^^27^^)^. The increase in liver fat deposition in fish fed the 2 g/kg diet was reflected by an increase in the NL content. In agreement with this observation, fatty liver is considered to be a common sign of EFA deficiency in salmonids^(^^28^^)^. Liver TAG content has been shown to increase when Atlantic salmon are fed diets in which fish oil is highly replaced by VO^(^^29^^–^^32^^)^. A recent study revealed that liver lipids increased with decreasing dietary levels of EPA and DHA (from 5·2 to 1·4 % of fatty acids; 17 to 5 g/kg feed) when the fish were reared at 6°C, whereas this effect was not evident at 12°C^(^^33^^)^. Adverse environmental conditions are known to exacerbate the effects of suboptimal dietary levels of key nutrients, which might lead to nutritional pathologies.

In the present study, EPA and DHA were found to gradually decrease in erythrocytes of fish fed decreasing levels of these fatty acids. In humans, low levels of *n*-3 VLC-PUFA in erythrocytes are considered a cardiovascular risk factor^(^^34^^)^. Therefore, reduced *n*-3 VLC-PUFA in fish erythrocytes might be an important welfare indicator. The decrease in EPA and DHA was compensated by an increase in *n*-6 PUFA, especially 18 : 2*n*-6, 20 : 3*n*-6 and 20 : 4*n*-6. Dietary levels of 20 : 4*n*-6 were similar among the fish fed the different experimental diets, indicating that *n*-6 elongation and desaturation activity was increased in fish fed the 2 and 10 g/kg diets. However, the *n*-6:*n*-3 ratio in erythrocytes from fish fed reduced levels of EFA was not as affected as that in the liver, indicating selective conservation of *n*-3 fatty acids.

PL are the fundamental components of cellular membranes, and changes in their fatty acid composition might affect the biophysical properties of membranes compromising a different array of essential metabolic processes^(^^35^^)^. The main fatty acids characterising the different PL classes were in agreement with those described before^(^^36^^,^^37^^)^. In addition, in accordance with literature reports^(^^36^^,^^38^^)^, the PC fraction was the most influenced by the diet. A significant increase in 18 : 2*n*-6 and its elongation products 20 : 3*n*-6 and 20 : 4*n*-6 was noted in the PC fraction of the skin and intestine in fish fed the 2 g/kg diet. These fatty acids were also enriched in the PC fraction of the muscle and heart in Atlantic salmon fed VO-based diets compared with those fed fish oil-rich diets^(^^38^^,^^39^^)^. In this study, the EPA tissue levels were relatively conserved in all PL classes except in PC, which was, as already mentioned, modulated by the diet to a larger extent. In contrast, DHA markedly decreased in all PL classes when the fish were fed diets containing reduced EFA content, with the exception of relative steady levels in the skin PI fraction and intestinal PE. This reduction in DHA was generally compensated by an increase in *n*-6 fatty acids, particularly 20 : 4*n*-6, and this increase was especially pronounced in the skin PE fraction and intestinal PI fraction. Since 20 : 4*n*-6 is the primary precursor of pro-inflammatory eicosanoids, its remarkable increase in the different PL classes might result in increased inflammation risk. If inflammation becomes chronic, tissue damage might become pathological, as is noted in a range of inflammatory conditions in humans^(^^40^^)^. In addition, intestinal changes in PL might affect lipoprotein synthesis and secretion, leading to impaired transport of lipid nutrients to storage tissues, as revealed by the high levels of fat accumulated in the intestine and liver of the fish fed the diet with low dietary levels of EFA.

Histological examination of the mid-intestine showed no specific pathology in the mucosa or associated structures, in particular, no signs of a widespread inflammatory reaction. In contrast, visual inspection of the mid-intestinal mucosae according to the BioMar scoring system showed red and swollen mucosae, which was interpreted as inflammation. The macroscopic visual scores differed significantly between diets and were clearly related to dietary EPA and DHA content. However, the lack of any corresponding difference in intestinal fold height, measured morphometrically, suggested that the visual observations were not caused by an inflammatory response, since intestinal inflammation would probably impair intestinal fold regeneration^(^^41^^)^. The histological observations of increased mucosal vacuolisation followed a pattern that corresponded better to the visually observed redness and swollenness, and the correlation between the macroscopic and microscopic observations were confirmed statistically. Thus, we infer that the vacuolisation could probably be attributed to lipid accumulation, which was observed macroscopically as swollen and red mucosae. The intestinal histology was considerably similar to that described in a group of human genetic disorders associated with lipid malabsorption, in which the lipidic nature of vacuolisation was the key finding^(^^42^^)^. We speculated that the fish showed compromised intestinal lipid absorption, which was visible in histological sections as enterocyte vacuolisation and on macroscopic examination as intestinal mucosal redness and swollenness. If our assumption is correct, the lipid malabsorption would be related to EPA and DHA insufficiency in the 2 and 10 g/kg diets. This finding is in agreement with that of Bou *et al.*^(^^3^^)^, who reported similar observations in smaller fish (400 g) fed diets low in EPA and/or DHA.

The most prominent X-ray examination finding was the high incidence of lack of normal intervertebral space, which was inversely correlated with dietary EPA and DHA content. A similar type of vertebral deformity was reported by Witten *et al.*^(^^22^^)^ with a suggested aetiology of early platyspondylia development. Such missing intervertebral spaces were predominantly located in the anterior spine in our study, unlike the predilected location of platyspondylia in the caudal spine. Further, vertical shift between adjacent vertebrae was found, which is not common in the early stages of platyspondylia, and individual vertebrae showed no signs of compression. Further, the lesions had a moderate and regular size, unlike the typical findings in platyspondylia. Thus, we hypothesised that our observation was of a different nature from that described by Witten *et al.*^(^^22^^)^. However, whether these lesions are related to reduced mineralisation is not yet known. The reduced vertebral length might indicate such a relationship, but the variation in vertebral length was smaller than the major differences between diet groups in fish with missing intervertebral space. A previous study performed in Atlantic salmon indicated that mineral content of the vertebrae might be affected by the dietary fatty acid composition, especially during the period prior to smoltification^(^^16^^)^. In rats, an increased *n*-6:*n*-3 PUFA ratio had a negative effect on bone formation^(^^43^^)^. In Atlantic salmon, an increase in *n*-6:*n*-3 PUFA ratio from 0·2 (FO diet) to 6 (VO diet) did not influence the development of inflammation-related spinal deformities^(^^44^^)^. However, both dietary treatments used in that study contained fish meal, which even in the VO-based diet fulfilled the *n*-3 VLC-PUFA requirements (22 g/kg feed). Alternatively, the high incidence of lack of normal intervertebral space in salmon fed the two lowest levels of EPA + DHA might be related to deficiency in the amount, function or strength of the connective tissue. In mammals, the therapeutic potential of EPA and DHA for preventing cartilage degeneration has been reported^(^^45^^–^^47^^)^. Specific pathological lesions in the vertebrae were detected in 18 % of fish, but no significant relation to diet was found, either in the incidence or extent of the lesions. The vertebral pathology types recorded were fusions and platyspondylia, both of which are typical observations in commercially produced salmon, and thus might not be linked to dietary contents of EPA and DHA. However, clinical diagnostics of spine deformities are little developed in salmon and further research is needed.

In conclusion, EPA and DHA levels of 10 g/kg in Atlantic salmon diets, a level previously regarded as sufficient, was found to be too low to maintain fish health or robustness under demanding environmental conditions in sea cages.

## Supplementary material

For supplementary material accompanying this paper visit http://dx.doi.org/10.1017/jns.2017.28. click here to view supplementary material
