# Supplementary material for: Low levels of very-long-chain n-3 PUFA in Atlantic salmon (Salmo salar) diet reduce fish robustness under challenging conditions in sea cages
Source: J Nutr Sci. 2017 Jun 28;6:e32. doi: 10.1017/jns.2017.28 (PMC5672314; doi:10.1017/jns.2017.28)
Supplement: Supplementary file 1 [file S2048679017000283sup.zip › S2048679017000283sup002.doc]

**Supplementary Table S2.** Significance levels of the one-way ANOVA testing effects of the different experimental diets on the fatty acid composition of the different phospholipid classes (phosphatidylcholine (PC), phosphatidylethanolamine (PE), phosphatidylinositol (PI), and phosphatidylserine (PS)) in skin and intestine. Differences between diets were determined by Tukey’s honest significant difference test (*P* < 0.05)

|  | PC | |  | PE | |  | PS | |  | PI | |
| --- | --- | --- | --- | --- | --- | --- | --- | --- | --- | --- | --- |
| Skin | Diet Effect | ANOVA |  | Diet Effect | ANOVA |  | Diet Effect | ANOVA |  | Diet Effect | ANOVA |
| 16:0 | 2 ≤ 17 ≤ 10 | 0.04 |  | 2 < 10 = 17 | 0.0004 |  | 2 = 10 = 17 | 0.15 |  | 2 = 10 = 17 | 0.96 |
| 18:0 | 2 > 10 > 17 | <0.0001 |  | 2 = 10 > 17 | 0.0003 |  | 2 ≥ 10 ≥ 17 | 0.01 |  | 2 = 10 = 17 | 0.56 |
| 18:1 n-9 | 2 > 10 = 17 | <0.0001 |  | 2 = 10 = 17 | 0.75 |  | 2 = 10 = 17 | 0.93 |  | 2 = 10 = 17 | 0.10 |
| 18:2 n-6 | 2 > 10 > 17 | <0.0001 |  | 2 > 10 > 17 | <0.0001 |  | 2 = 10 > 17 | <0.0001 |  | 2 = 10 = 17 | 0.16 |
| 20:3n-6 | 2 > 10 > 17 | <0.0001 |  | 2 > 10 > 17 | <0.0001 |  | 2 > 10 = 17 | <0.0001 |  | 2 = 10 = 17 | 0.88 |
| 20:4 n-6 | 2 > 10 > 17 | <0.0001 |  | 2 > 10 > 17 | <0.0001 |  | 2 > 10 = 17 | 0.005 |  | 2 = 10 = 17 | 0.65 |
| 20:5 n-3 | 2 < 10 < 17 | <0.0001 |  | 2 < 10 = 17 | 0.002 |  | 2 = 10 < 17 | <0.0001 |  | 2 = 10 = 17 | 0.26 |
| 22:5 n-3 | 2 < 10 < 17 | <0.0001 |  | 2 = 10 = 17 | 0.13 |  | 2 = 10 = 17 | 0.21 |  | 2 = 10 = 17 | 0.63 |
| 22:6 n-3 | 2 < 10 < 17 | <0.0001 |  | 2 < 10 < 17 | <0.0001 |  | 2 < 10 < 17 | <0.0001 |  | 2 = 10 = 17 | 0.07 |
| **Intestine** |  |  |  |  |  |  |  |  |  |  |  |
| 16:0 | 2 = 10 > 17 | <0.0000 |  | 2 = 10 = 17 | 0.43 |  | 2 = 10 = 17 | 0.06 |  | 2 = 10 = 17 | 0.38 |
| 18:0 | 2 > 10 > 17 | <0.0001 |  | 2 ≥ 10 ≥ 17 | 0.008 |  | 2 > 10 > 17 | <0.0001 |  | 2 > 10 > 17 | <0.0001 |
| 18:1 n-9 | 2 > 10 = 17 | <0.0001 |  | 2 = 10 = 17 | 0.91 |  | 2 = 10 = 17 | 0.91 |  | 2 = 10 = 17 | 0.21 |
| 18:2 n-6 | 2 > 10 > 17 | <0.0001 |  | 2 ≥ 10 ≥ 17 | 0.003 |  | 2 = 10 > 17 | 0.005 |  | 2 > 10 > 17 | <0.0001 |
| 20:3n-6 | 2 > 10 > 17 | <0.0001 |  | 2 > 10 > 17 | <0.0001 |  | 2 > 10 > 17 | <0.0001 |  | 2 > 10 > 17 | <0.0001 |
| 20:4 n-6 | 2 > 10 > 17 | <0.0001 |  | 2 > 10 > 17 | <0.0001 |  | 2 > 10 > 17 | <0.0001 |  | 2 > 10 > 17 | <0.0001 |
| 20:5 n-3 | 2 < 10 < 17 | <0.0001 |  | 2 = 10 = 17 | 0.21 |  | 2 = 10 = 17 | 0.35 |  | 2 ≤ 17 ≤ 10 | 0.02 |
| 22:5 n-3 | 2 < 10 < 17 | <0.0001 |  | 2 = 10 = 17 | 0.40 |  | 2 ≥ 10 ≥ 17 | 0.01 |  | 2 = 10 = 17 | 0.08 |
| 22:6 n-3 | 2 < 10 < 17 | <0.0001 |  | 2 = 10 = 17 | 0.24 |  | 2 < 10 < 17 | <0.0001 |  | 2 < 10 < 17 | <0.0001 |

In the diet effect columns, the numbers refer to the EPA and DHA content in the experimental diets expressed in g/kg. Thus, 2 refers to 2 g/kg EPA and DHA, 10 refers to the 10 g/kg EPA and DHA diet, and 17 refers to 17 g/kg EPA and DHA diet.
