# Supplementary material for: Low levels of very-long-chain n-3 PUFA in Atlantic salmon (Salmo salar) diet reduce fish robustness under challenging conditions in sea cages
Source: J Nutr Sci. 2017 Jun 28;6:e32. doi: 10.1017/jns.2017.28 (PMC5672314; doi:10.1017/jns.2017.28)
Supplement: Supplementary file 1 [file S2048679017000283sup.zip › S2048679017000283sup001.doc]

**Supplementary Table S1.** Amino acid content (%) of the experimental diets (5 mm)

| Amino acids (%) | 2 g/kg EPA+DHA | 10 g/kg EPA+DHA | 17 g/kg EPA+DHA |
| --- | --- | --- | --- |
| Arginine | 2.68 | 2.56 | 2.09 |
| Histidine | 1.15 | 1.15 | 1.10 |
| Isoleucine | 1.76 | 1.75 | 1.72 |
| Leucine | 3.17 | 3.16 | 3.19 |
| Lysine | 3.17 | 3.10 | 3.34 |
| Methionine | 0.92 | 0.93 | 0.97 |
| Phenylalanine | 1.91 | 1.88 | 1.95 |
| Threonine | 1.37 | 1.49 | 1.58 |
| Tryptophan | 0.37 | 0.39 | 0.39 |
| Valine | 1.83 | 1.83 | 1.75 |
| Alanine | 1.86 | 1.89 | 1.68 |
| Aspartic acid | 3.73 | 3.65 | 3.02 |
| Glutamic Acid | 6.28 | 6.15 | 8.23 |
| Glycine | 2.13 | 2.14 | 1.65 |
| Hydroxyproline | 0.33 | 0.33 | 0.10 |
| Proline | 2.19 | 2.39 | 2.78 |
| Serine | 1.95 | 1.93 | 1.91 |
| Tyrosine | 1.10 | 1.12 | 1.12 |
